# Identification of new genotype of Echovirus 19 from children with Acute Flaccid Paralysis in Pakistan

**DOI:** 10.1038/srep17456

**Published:** 2015-12-08

**Authors:** Mehar Angez, Shahzad Shaukat, Rabaab Zahra, Salmaan Sharif, Muhammad Masroor Alam, Adnan Khurshid, Muhammad Suleman Rana, Syed Sohail Zahoor Zaidi

**Affiliations:** 1 Department of Virology, National Institute of Health, Chak Shahzad, Park Road, Islamabad-45500, Pakistan; 2 Department of Microbiology, Faculty of Biological Sciences, Quaid-i-Azam University, Islamabad-45320, Pakistan

## Abstract

Enteroviruses are known to cause childhood paralysis. The purpose of this study was to examine the genetic diversity and to determine the association of non-polio enteroviruses (NPEVs) with acute flaccid Paralysis (AFP). Stool samples (n = 1191) of children with AFP were collected from Khyber Pakhtunkhwa and Federally Administered Tribal Areas of Pakistan. Poliovirus was isolated in 205 (17.2%) samples and NPEVs were found in 215 (18.0%) samples. Out of 215 viruses, 124 (57.7%) were typed into 19 different types of enteroviruses while 91 (42.3%) remained untypeable on microneutralization assay that were reconfirmed as NPEVs by real time PCR. Echovirus 19 (20/35; 57.1%) was found the most prevalent type based on VP1 nucleotide sequencing with increased genetic diversity. Phylogenetic analysis revealed the circulation of a new genotype of E-19 in the country. The findings of this study are of great importance for future research and propose to establish the enterovirus surveillance system in the country to readily identify more enteroviruses and to monitor the emergence of new variants/genotypes especially at the moment when we are at the verge of polio eradication phase.

Enteroviruses (EVs) are small (22 to 30 nm) non-enveloped, single-stranded RNA viruses comprising four viral capsid proteins VP1–4^1^. Traditionally, enteroviruses are classified into four groups (i) polioviruses (ii) coxsackieviruses A (iii) coxsackieviruses B (iv) enteric cytopathogenic human orphan (ECHO) viruses based on pathogenesis of infection in humans and experimental suckling mice. Later on, neutralization with type specific antisera was used for classification of these viruses^2^,^3^ but this approach failed to provide consistent results due to low sensitivity of virus isolation and emergence of many untypable/new types^4^,^5^. As a result, a molecular approach targeting VP1 encoding region was introduced for characterization of enteroviruses and it was proposed that EVs shall be classified as the same type if they share >75% nucleotide (>85% amino acid) identity in the VP1 coding sequence and into a new type if the nucleotide similarity is <75% with existing types^1^,^6^,^7^.

However, at present, based on the phylogenetic relationships in multiple genome regions, International Committee on Taxonomy of Viruses (ICTV) reclassified enteroviruses into twelve species, i.e., EV-A to H, EV-J and human rhinovirus (HRV)-A to C^8^,^9^,^10^. Only seven species (EV-A to D and HRV-A to C) infect humans while remaining infect cattle (bovine enteroviruses, porcine enteroviruses), ape species and monkey (simian enteroviruses). The EV-B is most diverse species with at least 61 types, including coxsackievirus (Cox) B1-6, A9, Echovirus (E) E1–7, 9, 11–21, 24–27, 29–33, and EV-B 69, 73–75, 77–88, 93, 97–98, 100–101, 106–107, 110–111 and Simian agent 5 (SA5), which are closely related to simian enteroviruses^11^,^12^.

Most enteroviral infections are asymptomatic; however, sometimes they cause mild to severe illness such as aseptic meningitis, encephalitis conjunctivitis, myocarditis, poliomyelitis, neonatal enteroviral sepsis and acute flaccid paralysis^13^,^14^.

Echovirus 19 was first isolated from stool sample of a child suffering from diarrhea in 1955^15^ and later from cerebrospinal fluid (CSF) of a man with aseptic meningitis in 1959^16^. However, epidemic of E-19 infection among infants and children has been observed in Europe during 1974 and 1975^17^,^18^. Similarly, association of this virus with other infections such as epidemic neuromyasthenia, uveitis, gastroenteritis and myalgia has been reported in previous years^19^ and recently echovirus 19 has been isolated from a case of AFP child in Australia^20^.

In this study, we analyzed stool samples collected from acute flaccid paralytic children residing in Khyber Pakhtunkhwa (KP) and Federally Administered Tribal Areas (FATA) of Pakistan to investigate the association of NPEVs with AFP and to examine the genetic diversity among these viruses. The genetic data reveals that echovirus 19 is the most prevalent serotype and has some possible link in causation of paralysis. Furthermore, genome analysis confirms the circulation of a new indigenous genotype of E-19 with increased genetic diversity in the country.

## Results

### Enterovirus Cell Culture and Microneutralization

A total of 1191 stool samples (887 from Khyber Pakhtunkhwa and 304 from FATA; each represents a unique patient) collected during January to December 2013 from AFP patients were processed and inoculated on RD and L20B cell lines. Poliovirus was detected in 205 (17.2%) samples and non polio enteroviruses were found in 215 (18.0%) samples (Table 1). Out of 215 samples (n = 45 from FATA and n = 170 from KP) 124 (57.7%) samples were successfully typed into 19 different enterovirus serotypes (E-1-7, 11-14, 20, 21, 25, 27, 29, 30, 33 and CV-A9) while 91 (42.3%) remained untypeable by microneutralization assay.

### Molecular Typing

All untypeable isolates (n = 91) were reconfirmed as NPEVs by real-time reverse transcriptase polymerase chain reaction targeting 5′-UTR^21^ and nucleotide sequencing of a partial VP1 specific fragment^22^ placed 97.8% of these isolates into EV-B and 2.2% into EV-C species (data not shown). It has been found that majority (35/89; 39.3%) were echoviruses with more prevalent type Echovirus 19 (20/35; 57.1%). The clinical features of patients having AFP associated with E-19 are shown in Table 2.

### VP1 sequence analysis of Pakistan Echovirus 19 isolates

To investigate the molecular epidemiology and genetic diversity of most prevalent serotype E-19, partial VP1 nucleotide sequences (399 base pairs) of all 20 isolates were compared with prototype sequences of different enteroviruses retrieved from GenBank. All study strains (n = 20) were found closely related with echovirus 19 prototype (Burke; Accession No. AF081332) having mean 73.7% (73.4–76.9%) nucleotide identity within enterovirus B species and fulfills the new genotype demarcation criterion as described earlier^5^,^23^,^24^,^25^,^26^,^27^ that strain sharing <85% nucleotide identity in VP1 region with prototype sequence should be placed in different genotype. Therefore, it is proposed that Pakistan (PAK) isolates form a new genotype of E-19.

### Genetic Diversity among Pakistan Echovirus 19 isolates

**Figure 1:**
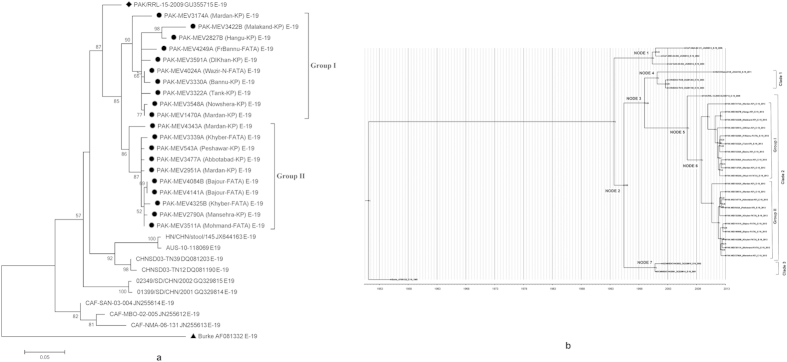
(**a**) A Phylogenetic tree was constructed from the partial VP1 nucleotide sequences (399 nucleotides) of twenty echovirus 19 strains and their closely related sequences retrieved from GenBank (accession numbers are included in the virus names) using maximum likelihood method with 1000 bootstrap by MEGA 5.0. Bootstrap values greater than 50 are indicated at the respective nodes and the scale bar represents the evolutionary distance. Genotype and groups are marked on the tree. The isolates from this study, closely related and prototype strains are represented by ‘●’, ‘♦’ and ‘▲’ taxon markers respectively. (**b**) Maximum clade credibility (MCC) tree based on the Bayesian analysis of the partial VP1 nucleotide sequences of PAK E-19 and their closely related sequences retrieved from GenBank. The tree was estimated with Hasegawa-Kishino-Yano nucleotide substitution model under the uncorrelated relaxed clock approach (UCLD). Branch tips correspond to date of collection and each tip represents a virus strain in 1 patient. Tree nodes were annotated with posterior probability (pp) values. Horizontal axis indicates time in years. PAK: Pakistan.

Phylogenetic trees were constructed by aligning the partial VP1 sequences of E-19 study strains with their closely related sequences retrieved from GenBank to study their genetic diversity and possible relationships. Pair wise distance scores confirmed that all study isolates constitute a separate clade (bootstrap 85%) that further segregated into two distinct genetic groups (I and II) with 93.3% (89.7–94.8%) mean nucleotide identity with each other supported by high bootstrap value 90 and 86 respectively (Fig. 1a).

Group I comprised 10 isolates from different districts of KP and FATA showing 73.7% (74.0–76.9%) mean nucleotide identity with E-19 prototype strain. All isolates in Group I presented 96.6% (91.7–98.9%) mean nucleotide identity with each other and one isolate PAK-MEV3422B isolated from district Malakand was most divergent isolate showing 95.14% (91.7–97.0%) mean nucleotide identity. Further it was observed that the closely related isolate to group I was PAK/RRL-15-2009 (Pakistan strain; Accession No. GU355715) showing 92.1% (87.9–93.8%) mean nucleotide identity.

Similarly, group II contained 10 isolates presenting 73.8% (72.7–75.1%) mean nucleotide identity with E-19 reference strain and 98% (95.4–99.6%) mean nucleotide identity among each other. The most divergent strain in this group was PAK-MEV4343A isolated from district Mardan with 96.1% (95.4–96.6%) mean nucleotide identity and it was also noticed that closely related strain to group II members was PAK/RRL-15-2009 having 92.4% (91.7–92.9%) mean nucleotide identity.

### Phylogenetic Analyses of Pakistan Echovirus 19 Isolates

The molecular evolution of PAK E-19 strains was also investigated by phylogenetic analysis together with closely related E-19 VP1 sequences from the GenBank database (Fig. 1a). The phylogenetic tree revealed that study strains of E-19 represent a clade which is completely different from those detected earlier from China, Australia and Central African Republic. All PAK study isolates had distinct genetic relationship with other E-19 strains and constitute a separate genetic clade representing a new genotype of E-19.

In addition, the partial VP1 gene sequences were also analyzed using a Bayesian method to estimate the phylogenetic tree topology, to investigate the genetic relationship of study isolates sequences and to classify them consistently among the current echovirus 19 strains. The Bayesian phylogram topology (Fig. 1b) was congruent with the topology previously observed in Maximum Likelihood tree (Fig. 1a). Overall, all the sequences of E-19 serotype sampled from 1955 to 2013 were distributed into two major partitions identified by ancestral nodes 1 and 2 (Fig. 1b). First partition contained only three isolates reported from Central African Republic. The second major tree partition was characterized by three different clades (1–3) with posterior probability 1. In this partition, a distinct clade 2 corresponding to Pakistan isolates clearly indicates a strong temporal clustering of sequences with two different groups supported by posterior probability 1 (Fig. 1b). Additionally, the study isolates displayed a close relatedness with an earlier identified E-19 PAK isolate (PAK-RRL-15-2009; Accession No. GU355715).

The most recent common ancestor of all PAK isolates dated back to about seven years ago 2006.3 (95% HPD: 2003.9–2008.4 years). Later on, the study isolates has diverged into two groups through node 6 (pp = 1) whose time was estimated to be 2008.7 (95% HPD: 2006.7–2010.5) years. Furthermore, group I was originated first as compared to Group II and the time origin of isolates into group I was estimated to be 2009.9 (95% HPD: 2008.2–2011.3) years while for Group II was 2010.5 (95% HPD: 2009.11–2011.7) years. The evolutionary timescale analysis of E-19 VP1 sequences revealed that the study strains diverged 58 years ago (1955) from their first isolated prototype (Burke). The tree root was estimated to the year 1949 with a credibility interval ranging between the years 1936.1 and 1955 (Fig. 1b).

### Amino acid Sequence Comparison

**Figure 2:**
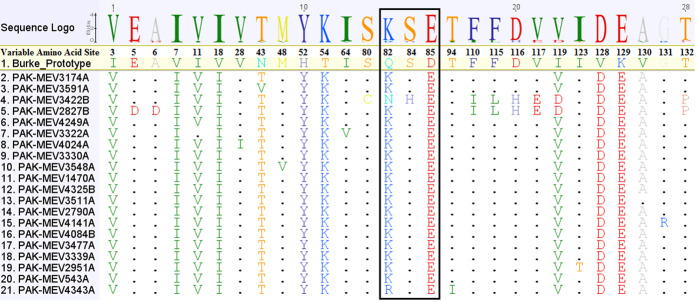
Alignment of deduced amino acid sequences of the VP1 polyprotein of echovirus 19 strains with the prototype sequence showing only the variable sites. The sequences within the rectangle show variable sites within the BC-loop region. Strain designations are on the left hand side of the alignment. Dots (.) represent amino acids identical with respect to prototype strain of echovirus 19 and the amino acid symbols indicate where unrelated amino acids exist with reference to prototype strain. In sequence logo the height of the logo at each site is equal to the total information at that site and the height of each symbol in the logo is proportional to its contribution to the information content.

The deduced amino acid sequences (133 amino acids) of VP1 region of all PAK E-19 strains compared with prototype Burke strain and similarity rate was observed 90.5% (88.5–91.8%). Amino acid alignments revealed that there were 105 (78.9%) conserved and 29 (21.1%) variable amino acid sites (Fig. 2). In addition, three substitutions were found in BC loop region (82 to 92 amino acid residues) between E-19 study strains and Burke prototype strains. Among these substitutions, one was glutamine (Q) to lysine (K) at amino acid 82 except in two isolates (PAK-MEV3422B and PAK-MEV4343A) where glutamine was replaced by Asparagine (N) and Arginine (R) respectively. The second substitution was serine (S) to histidine (H) at amino acid 84 and third was aspartic acid (D) to glutamic acid (E) at amino acid 85. No genotype specific amino acid signatures were observed in the sequence.

## Discussion

Acute flaccid paralysis (AFP) is a heterogeneous neurologic condition defined by the sudden onset of weakness and floppiness in any part of the body in a child <15 years of age or paralysis in a person of any age in whom poliomyelitis is suspected^28^.The predominant cause of AFP is infection with poliovirus but a number of non polio enteroviruses including coxsackieviruses, echoviruses and enterovirus 68, 70, 71 have also been reported to cause many neurological disorders such as encephalitis, meningitis, meningoencephalitis and AFP^29^,^30^,^31^,^32^,^33^. Similarly, in Pakistan many non polio enteroviruses have been isolated from AFP cases but unfortunately, these cases were never being explored further either by the polio eradication programme or the country health care system. As a result, no clear and detailed information about the epidemiology of NPEVs in the country is available except few reports^34^,^35^,^36^,^37^. Therefore, present study was designed to genetically characterize the NPEVs isolated from paralytic cases, as well as to understand the role of echovirus 19 in acute flaccid paralysis owing to the fact that Pakistan is at the verge to achieve the goal of polio eradication.

Echoviruses frequently cause aseptic meningitis^38^ but they may involve in paralysis, encephalitis, neonatal sepsis, exanthema, respiratory disease, myalgia, myocarditis, uveitis and hand-foot and mouth disease^19^,^39^. It was observed that proportion of echoviruses isolated in this study was high (73.1%) as compared to the numbered enteroviruses (21.7%) and coxsackieviruses (4.6%). Similarly, among echoviruses, the echovirus 19 was found the most prevalent human enterovirus (12.6%). Although echovirus 19 may cause mild to severe neurological disorders as reported from Malaysia, China, United States of America and Australia^20^,^40^,^41^,^42^,^43^ but unfortunately, no clinical information, molecular epidemiology and disease correlation of this serotype with AFP have been reported except two case reports. In first case, E-19 was isolated from CSF sample of a 39 year old immunosuppressed transplant recipient who admitted to the hospital with acute onset of flaccid paralysis of the left leg^43^ and in second case a three years old Australian child admitted to hospital with a 2-day history of fever to 38.8 °C and a 24-h history of painless inability to move her left upper arm and shoulder. A detailed medical history showed weakness restricted to the left upper limb, with power at the shoulder and elbow. The muscle tone was flaccid, but there was some degree of wrist flexion and extension and preservation of finger movements, with an absence of biceps, triceps and brachioradialis reflexes. Her stool sample was processed for NPEVs and confirmed it as E-19^20^. On discharge from hospital she had physiotherapy and rehabilitation but had residual weakness of her upper limb after 6 months. This case report supports our current study finding that suggests E-19 may have contribution in paralysis. Although, NPEVs are rarely associated with sporadic cases of AFP but we have isolated twenty E-19 strains from paralytic patients reside in a small geographic area of Pakistan. Clinically, all these patients presented fever, asymmetrical paralysis and rapid progression of paralysis at the time of onset of the disease. Additionally, residual weakness was seen in a marked proportion of AFP cases on 60 days follow up investigation. Consequently, all these findings suggest E-19 as the possible causative agent of paralysis and to the best of our knowledge; this is first report about the epidemiological linkage of E-19 with AFP published from Pakistan so far.

Furthermore, all viruses of this study, isolated from AFP cases grouped together in E-19 cluster and form a distinct genotype having 26.3% nucleotide divergence from their prototype and fulfill the criterion for assessment of new genotype as described previously^5^,^23^,^24^,^25^,^26^,^27^,^36^,^44^,^45^. Moreover, it was perceived by Bayesian evolutionary analysis (Fig. 1b), that the most recent common ancestors for both groups 1 and 2 viruses were dated to 2008.7, indicating that the two groups of new E-19 genotype were circulated for ~4.5 years in the community and were not simply the result of any current transmission. The maximum clade credibility (MCC) tree was also notable for the presence of separate clade containing the study isolates that were shown to have diverged into two groups and been introduced from China in year 2006.2 in the country. Similarly, the frequent presence of long branches in the tree point out towards increased genetic diversity that indicates a gap, probably created by a small number of samples collected and analyzed during the study period.

The BC loop in the VP1 region is, in particular, one of the regions associated with viral antigenicity, and substitution mutations may result in changes in this region, can play a role in host adaptation for enteroviruses^46^. All PAK E-19 isolates had three substitutions within the BC loop on comparison with prototype. Therefore, the effect of these mutations may need further investigations to rule out any changes in antigenic and biochemical properties.

In conclusion, we presented a genetic overview of E-19 with increased genomic diversity and explored its possible association with acute flaccid paralysis. Although Pakistan is a polio endemic country where more efforts are focused on poliovirus eradication but there is a dire need to assess the impact of the disease burden caused by this group of viruses as polio vaccine offers no protection against NPEVs infection. Moreover, we speculate that NPEVs may predominate in its toll to cause paralysis after post-eradication. Therefore, enterovirus surveillance is needed to strengthen in the country in order to get more epidemiological and clinical information about NPEVs.

## Methods

### Ethical approval

This Study was approved by the Institutional Internal Review Committee. All experiments and methods were performed in accordance with approved guidelines of Internal Review Committee of National Institute of Health, Pakistan. Informed consent was obtained from the legal guardians of the children enrolled in this study.

### Samples collection

Stool samples of children with Acute Flaccid Paralysis (AFP) were collected from Khyber Pakhtunkhwa (KP) and Federally Administered Tribal Areas (FATA) of Pakistan as per WHO guidelines within 14 days from the date of onset. Samples were transported to WHO Regional Reference Laboratory for Polio Eradication Initiative, Department of Virology, National Institute of Health, Islamabad, Pakistan under cold conditions along with patient clinical history and demographic data. A 60-day follow-up investigation after the onset of paralysis was carried out in those patients who experienced residual paralysis or weakness in one or more limb/s.

### Virus Isolation

Stool samples were treated with chloroform in phosphate buffer saline solution (PBS) to remove bacteria and fungi. A total of 200 μl of Stool supernatant of each sample was inoculated on RD (derived from rabdomayosarcoma cells of humans) and L20B cell culture tubes (mouse lymphoma cells expressing the human poliovirus receptor) and incubated at 36 °C^47^,^48^. A daily microscopic observation of each cell culture tube was recorded for characteristic enterovirus cytopathic effect (CPE) and samples positive on RD cells but negative on L20B cells was re-passaged on L20B cell to rule out the presence of polioviruses.

### Microneutralization Assay

Microneutralization assay was performed in 96-well tissue culture plates by using typing sera kit provided by National Institute for Public Health and the Environment (RIVM), Bilthoven^47^.

### RNA Extraction, Enterovirus Detection by qRT-PCR and VP1 R T-PCR Amplification

RNA was extracted from the supernatant of infected cells using the QIAamp Viral RNA extraction Kit (Qiagen, Valencia, CA) as per the manufacturer’s instruction. Non polio enteroviruses (NPEVs) were confirmed by real time reverse transcription Polymerase Chain Reaction (qRT-PCR) targeting 5´ UTR^21^. All screened NPEVs were further characterized by sequencing of VP1 gene through RT-PCR. Initially, complementary DNA (cDNA) was synthesized in a 20 μL reaction mixture comprising of 11 μL of each viral RNA, 4 μL of 5x transcriptase buffer, 2 μL of 10 mM DTT, 0.5 μL of 25 mM each dNTP mix (Roche), 1 μL of 40U of RNase Inhibitor and 1 μL of 20U AMV reverse transcriptase (Thermo Scientific) at 42 °C for 45 min. For amplification of VP1 region RCR was performed by using 2 μL of cDNA, 5 μL of 10xTaq buffer, 3.5 μL of MgCl2 (50 mM), 1.0 μL of 12.5 mM dNTPs, 0.5 μL of Taq DNA polymerase (5 units/ μL), 37 μL of water and 0.5 μL of each primer (10 μM) 224–222 targeting VP3 (nt 2204–2223) and VP1 (nt 2969–2951) region of virus genome^49^. Thermocycler parameters for RT-PCR was 95 °C for 3 min followed by 40 cycles of 94 °C for 45 s, 42 °C for 45 s, and 72 °C for 1 min. Specific RT-PCR products were purified using the Qiaquick PCR purification kit (Qiagen) and stored at −20 °C till further procedure.

### Nucleotide Sequencing and Phylogenetic analysis

The purified RT-PCR products were sequenced with overlapping primer set 224–222^22^ using a PRISM Big Dye Terminator cycle sequencing reaction kit version 3.1 (Perkin Elmer Applied Biosystem). Sequence data was generated by an automated ABI 3130 Genetic Analyzer and sequences were assembled and edited by using Sequencher 4.9 software (GeneCodes) (http://www.genecodes.com).

Genetic differences among E-19 isolates were analyzed by MEGA 5.0 software using partial (399 bp) VP1 region^50^. Sequence of E-19 prototype and all the representative strains retrieved from GenBank (http://www.ncbi.nlm.nih. gov/BLAST^51^ and aligned using ClustalW^52^. Evolutionary distances have been computed by using Maximum Composite Likelihood model and Jones–Thornton–Taylor (JTT) model for nucleotide and deduced amino acid sequences respectively^53^. Phylogenetic tree was constructed by maximum-likelihood method^54^ with 1000 bootstrap replicates^55^,^56^.

The evolutionary history of Echovirus 19 dataset was estimated by using a Bayesian Markov chain Monte Carlo (MCMC) approach as implemented in the Bayesian Evolutionary Sampling Tree (BEAST) package (v1.8.1; http://beast.bio.ed.ac.uk/Beast)^57^. The Hasegawa-Kishino-Yano (HKY) + Gamma distribution (G) was the best fitting model of evolution as estimated by jModelTest 2.1.7^58^,^59^. Relaxed molecular clock model (the uncorrelated lognormal-distributed model [UCLD]) was employed. MCMC analyses were run for 20 million generations, sampling a tree for every 1000 steps and the first 10% were discarded as burn-in. MCMC convergence and effective sample size (ESS) estimates were checked with Tracer (v1.6.0; http://beast.bio.ed.ac.uk/Tracer). A maximum clade credibility (MCC) tree was obtained using TreeAnnotator program (v1.8.1; http://beast.bio.ed.ac.uk/TreeAnnotator) and visualized in FigTree (v1.4.2; http://beast.bio.ed.ac.uk/figtree). Time to the most recent common ancestor (tMRCA) at each node in the phylogeny was calculated from the height value in MCC tree and 95% highest posterior density (HPD) interval was estimated.

## Additional Information

**Accession codes**: VP1 sequences of echovirus 19 strains identified in this study were deposited in GenBank under accession number KP814112 to KP814131.

**How to cite this article**: Angez, M. *et al*. Identification of new genotype of Echovirus 19 from children with Acute Flaccid Paralysis in Pakistan. *Sci. Rep*. **5**, 17456; doi: 10.1038/srep17456 (2015).

## Figures and Tables

**Table 1:** Isolation of non-polio Enterovirus from AFP patients in Cell Culture.

Region	Cytopathic Effect					
	RD Cell Line		L20B Cell Line		RD −>L20BCell Line	
					n = 420*	
	Positive	Negative	Positive	Negative	Positive	Negative
KP n = 887	236	651	66	821	66	170
FATA n = 304	184	120	139	165	139	45
Total n = 1191	420	771	205	986	205	215

**Table 2:** Clinical and Demographic data of Acute Flaccid Paralytic patients infected with E19.

S.No	Lab.No.	Region	District	Sample collection date	Sex (male/female)	Age(months)	Fever	Asymmetric paralysis	Progression of paralysis
1	PAK-MEV543	KP	Peshawar	16-Feb-2013	M	12	Yes	Yes	Yes
2	PAK-MEV1470A	KP	Mardan	23-Apr-2013	M	18	No	No	Yes
3	PAK-MEV2790A	KP	Mansehra	4-Jul-2013	F	18	No	Yes	Yes
4	PAK-MEV2827B	KP	Hangu	9-Jul-2013	M	12	Yes	Yes	Yes
5	PAK-MEV2951A	KP	Mardan	16-Jul-2013	M	23	Yes	Yes	Yes
6	PAK-MEV3174A	KP	Mardan	28-Jul-2013	M	2	Yes	No	Yes
7	PAK-MEV3322A	KP	Tank	7-Aug-2013	M	36	Yes	No	Yes
8	PAK-MEV3330A	KP	Bannu	11-Aug-2013	F	13	No	No	Yes
9	PAK-MEV3339A	FATA	Khyber	6-Aug-2013	M	18	Yes	Yes	Yes
10	PAK-MEV3422B	KP	Malakand	15-Aug-2013	F	22	No	No	Yes
11	PAK-MEV3477A	KP	Abotabad	16-Aug-2013	M	49	No	No	Yes
12	PAK-MEV3511A	FATA	Mohmand	20-Aug-2013	F	22	No	Yes	Yes
13	PAK-MEV3548A	KP	Nowshera	21-Aug-2013	M	18	Yes	No	Yes
14	PAK-MEV3591A	KP	DI Khan	24-Aug-2013	M	12	No	Yes	Yes
15	PAK-MEV4024A	FATA	Wazir-N	12-Sep-2013	F	7	Yes	Yes	Yes
16	PAK-MEV4084B	FATA	Bajour	16-Sep-2013	F	48	No	No	Yes
17	PAK-MEV4141A	FATA	Bajour	17-Sep-2013	M	60	Yes	Yes	Yes
18	PAK-MEV4249A	FATA	FR Bannu	22-Sep-2013	M	9	Yes	Yes	Yes
19	PAK-MEV4325B	FATA	Khyber	25-Sep-2013	M	8	Yes	Yes	Yes
20	PAK-MEV4343A	KP	Mardan	27-Sep-2013	M	45	Yes	Yes	Yes
